# GABA-B Agonist Baclofen Normalizes Auditory-Evoked Neural Oscillations and Behavioral Deficits in the *Fmr1* Knockout Mouse Model of Fragile X Syndrome

**DOI:** 10.1523/ENEURO.0380-16.2017

**Published:** 2017-03-01

**Authors:** D. Sinclair, R. Featherstone, M. Naschek, J. Nam, A. Du, S. Wright, K. Pance, O. Melnychenko, R. Weger, S. Akuzawa, M. Matsumoto, S. J. Siegel

**Affiliations:** 1 Translational Neuroscience Program Department of Psychiatry, University of Pennsylvania, Philadelphia, PA 19104, USA; 2 Neuroscience Research Unit, DDR, Astellas Pharma Inc., Tsukuba-Shi, Ibaraki 305-8585, Japan

**Keywords:** EEG, fragile X syndrome, GABA, gamma, racemic baclofen, working memory

## Abstract

Fragile X syndrome is a genetic condition resulting from *FMR1* gene mutation that leads to intellectual disability, autism-like symptoms, and sensory hypersensitivity. Arbaclofen, a GABA-B agonist, has shown efficacy in some individuals with FXS but has become unavailable after unsuccessful clinical trials, prompting interest in publicly available, racemic baclofen. The present study investigated whether racemic baclofen can remediate abnormalities of neural circuit function, sensory processing, and behavior in *Fmr1* knockout mice, a rodent model of fragile X syndrome. *Fmr1* knockout mice showed increased baseline and auditory-evoked high-frequency gamma (30–80 Hz) power relative to C57BL/6 controls, as measured by electroencephalography. These deficits were accompanied by decreased T maze spontaneous alternation, decreased social interactions, and increased open field center time, suggestive of diminished working memory, sociability, and anxiety-like behavior, respectively. Abnormal auditory-evoked gamma oscillations, working memory, and anxiety-related behavior were normalized by treatment with baclofen, but impaired sociability was not. Improvements in working memory were evident predominantly in mice whose auditory-evoked gamma oscillations were dampened by baclofen. These findings suggest that racemic baclofen may be useful for targeting sensory and cognitive disturbances in fragile X syndrome.

## Significance Statement

Baclofen stimulates the inhibitory GABA-B receptor and is the publicly available version of arbaclofen, a candidate drug for fragile X syndrome that showed initial promise in clinical trials but did not succeed overall and is now unavailable. Using the *Fmr1* knockout mouse model of fragile X syndrome, we show that baclofen may have potential for targeting abnormal sensory sensitivity (in the form of increased auditory-evoked high-frequency neural oscillations) and cognitive deficits (particularly spatial working memory) in fragile X syndrome. This work also highlights the possible usefulness of electroencephalography, which measures neural oscillations, for predicting treatment responsiveness in select fragile X symptom domains.

## Introduction

Fragile X syndrome (FXS) is a debilitating neurodevelopmental disorder that results from mutation of the *FMR1* gene (Verkerk et al. 1991) and loss of fragile X mental retardation protein (FMRP). It affects ∼1 in 4000 males and 1 in 8000 females (Turner et al. 1996) and is characterized by cognitive deficits, hyperactivity, attention problems, social communication deficits, and increased anxiety (Bailey et al. 2008). Approximately 40% of individuals with FXS display symptoms of autism spectrum disorder (ASD; Bailey et al. 2008). Treatment options for FXS are currently limited and have historically focused on symptoms such as anxiety (Bailey et al. 2012). More recently, evidence-based FXS pharmacotherapies (Healy et al. 2011; Lozano et al. 2014) have emerged. Some such therapies have targeted the γ-aminobutyric acid (GABA) neurotransmitter signaling pathway, based on evidence of GABAergic deficits in *Fmr1* knockout (KO) mice (Gantois et al. 2006; Adusei et al. 2010; Paluszkiewicz et al. 2011), a rodent model with strong construct validity for FXS.

The GABA-B agonist arbaclofen (STX209) has shown promise preclinically, rescuing behavioral abnormalities in *Fmr1* KO mice (Henderson et al. 2012; Qin et al. 2015) and mouse models of ASD (Silverman et al. 2015). Studies on the effects of arbaclofen in individual with FXS have shown mixed results (Lozano et al. 2014). In adults and children with FXS, arbaclofen did not improve overall symptom severity (Berry-Kravis et al. 2012). However, improvement in select symptom domains was seen in some individuals, consistent with anecdotal evidence of efficacy provided by families of individuals with FXS (Lozano et al. 2014). In the face of these results, arbaclofen trials in FXS were ceased in May 2015, and the drug was made unavailable for clinical research.

The overall failure of arbaclofen in human FXS trials may have occurred because arbaclofen is beneficial for only a subset of individuals or is effective for only a limited subset of symptoms. Furthermore, although arbaclofen is currently unavailable for research or treatment, racemic baclofen is available and may be used off-label for FXS. As a result, it is critical to establish the usefulness of racemic baclofen for treating key FXS symptom domains and behaviors.

Sensory abnormalities are key symptoms of FXS and ASD (Sinclair et al. 2016) that may differentiate individual responses to baclofen. Sensory abnormalities can be objectively assessed using electroencephalography (EEG) in both humans with FXS and *Fmr1* KO mice. Humans with FXS show evidence of auditory hypersensitivity, disrupted preattentive stimulus recognition, and altered novelty detection by EEG (St Clair et al. 1987; Castrén et al. 2003; Van der Molen et al. 2012a, b; Ethridge et al. 2016), as do *Fmr1* KO mice (Chen and Toth, 2001; Frankland et al. 2004; Gibson et al. 2008; Lovelace et al. 2016). Because abnormalities of sensory processing are likely to contribute to sensory hypersensitivity and other deficits in FXS, they are an important potential target for baclofen treatment.

Therefore, we had three primary aims. First, we set out to determine the extent of sensory processing abnormalities in *Fmr1* KO mice, particularly at the level of *in vivo* neural oscillations. Although EEG has been used previously in *Fmr1* KO mice (Gonçalves et al. 2013; Radwan et al. 2016), to our knowledge auditory-evoked oscillations have not been investigated. Second, we explored whether EEG oscillation abnormalities and behavioral deficits in *Fmr1* KO mice could be rescued by acute racemic baclofen treatment. *Fmr1* KO mice show abnormal locomotor activity, anxiety-like behavior, reduced social interactions, and impaired working memory (Bakker et al. 1994; Kramvis et al. 2013; Hébert et al. 2014; Oddi et al. 2015), but the amenability of these deficits to racemic baclofen treatment is unclear (Pacey et al. 2009). Finally, we investigated whether rescue of specific EEG oscillation abnormalities at an individual level was associated with improvement in behavior or cognition, as a proof of concept for the possible future use of EEG to predict treatment responsiveness in individuals with FXS.

## Materials and Methods

### Study design

This study compared inbred adult *Fmr1* KO mice to wild-type (WT) controls using a within-subject design, testing the hypothesis that sensory hypersensitivity in *Fmr1* KO mice would be reflected in abnormal stimulus-evoked neural activity and would relate to behavioral deficits. Neural oscillations (at baseline and in response to auditory stimulus), open-field behavior, social interactions, spatial working memory, and spatial episodic memory were quantified sequentially in the same mice. Each test was conducted on four separate days. Vehicle was administered on test day 1, then 1 mg/kg baclofen on test day 2, 2.5 mg/kg baclofen on test day 3 and 5 mg/kg baclofen on test day 4, with 48 h washout between test days. Based on previous experience of the sample size required to reveal biologically relevant differences in EEG and behavior (Gandal et al. 2012a, b; Billingslea et al. 2014; Tatard-Leitman et al. 2015), 15–23 mice per genotype and sex were used.

### Animals and drug preparation

Homozygous female *Fmr1* KO mice and hemizygous male *Fmr1* KO mice on a C57BL/6 background (strain name B6.129P2-*Fmr1^tm1Cgr^*/J, stock no. 003025; RRID: MGI:4950026; Bakker et al. 1994), along with WT C57BL/6 controls, were received from The Jackson Laboratory at 28 d of age. Littermates did not serve as WT controls, since the required breeding strategy for homozygous female *Fmr1* KO mice does not generate WT mice. Animals were group-housed in standard cages (Allentown Inc.) with food and water *ad libitum,* in a room maintained on a 12 h light-dark cycle. Mice were habituated to the colony environment for 4 weeks before electrode implantation and underwent surgery at postnatal day ∼56. Animals were singly housed for the remainder of the study to prevent postoperative complications. All protocols for animal care and use were undertaken in accordance with University Laboratory Animal Resources and NIH guidelines and were approved by the Institutional Animal Care and Use Committee (University of Pennsylvania protocol #803572). Racemic baclofen (cat. #5399; Sigma-Aldrich) was dissolved in 0.9% saline immediately before use and administered intraperitoneally at 1, 2.5, or 5 mg/kg in 100 µl injection volume. Testing was conducted 5 min after injection.

### Electroencephalography

Animals were anaesthetized with isoflurane and implanted unilaterally with a tripolar electrode assembly. The recording electrode was placed 1.8 mm posterior, 2.65 mm right lateral, and 2.75 mm deep relative to Bregma to detect changes in the electrical field emanating from the hippocampus and cortex. Reference and ground electrodes were placed transversely onto the surface of the ipsilateral neocortex, 0.8 mm deep (Featherstone et al. 2015). Electrodes were secured to the skull with ethyl cyanoacrylate and dental cement. Mice were allowed a 10 d period of recovery before testing. Neural oscillations were recorded within a Faraday cage using a high-impedance differential AC amplifier (model #1800, A-M Systems) and Spike 2 software (Cambridge Electronic Design). Baseline oscillations were recorded in the first minute of the session, 5 min after IP injection. Thereafter, 120 broadband white-noise stimuli (10 ms duration, 85 dB with an 8 s interstimulus interval) were administered. Raw EEG was filtered between 1 and 500 Hz, and individual sweeps were rejected for movement artifact based on a criterion of 2× root mean squared amplitude per mouse. For event-related potentials (ERPs), the P20 peak was defined as the largest positive voltage deflection between 15 and 30 ms and the N40 peak as the largest negative voltage deflection between 30 and 100 ms. Baseline power was derived by fast Fourier transformation (FFT) using the FFT function native to Spike2. Baseline absolute gamma power was quantified as the average gamma power (30–80 Hz) across the 60 s prestimulus period. Baseline relative gamma power was expressed as the proportion of gamma power (30–80 Hz) relative to total power (0–120 Hz). Event-related power from 0 to 200 ms after stimulus was baseline-corrected and derived using time-frequency decomposition methods native to the EEGLAB toolbox (Delorme and Makeig, 2004; RRID: SCR_007292) within Matlab (Mathworks; RRID: SCR_001622) as previously described (Billingslea et al. 2014; Tatard-Leitman et al. 2015).

### Behavioral tests

Behavioral testing was performed with *Fmr1* KO and WT mice at 6–10 weeks of age. Testing took place during the light phase. Each test was repeated on four separate sessions for administration of vehicle or 1, 2.5, or 5 mg/kg baclofen, with a 48 h washout between sessions. There was a break of 4–5 days between each behavioral test. The chronological order of tests was open field, continuous T-maze, three-chamber social interaction, and modified radial water maze.

For the open-field test, mice acclimated in the testing room for 15 min before recording. Five minutes after IP injection with saline or baclofen, mice were placed in a clear Perspex open-field recording chamber (40 × 40 cm) divided into a 16 × 16 photobeam grid (Photobeam Activity System, San Diego Instruments). Locomotor activity (the number of photobeam breaks in 10 min), time spent in the center of the chamber (central area, 15 × 15 cm), and time spent in the periphery (peripheral area, 25 cm zone flanking chamber walls) were the measures of interest, quantified using Photobeam Activity System software (San Diego Instruments). Two mice at 5 mg/kg baclofen (one female WT and one female KO) were omitted because of technical problems recording the test.

The apparatus used for assessing social interaction consisted of two interaction chambers (19 cm wide, 26 cm deep, and 22 cm tall), each containing a wire mesh cylinder (8 cm in diameter, 12 cm tall) as previously described (Sankoorikal et al. 2006; Ehrlichman et al. 2009). The interaction chambers were separated by a central chamber (13 cm wide, 26 cm deep, and 22 cm tall). The walls between chambers had an opening 11 cm wide. The testing room was maintained under dim light. At the start of the test the mesh cylinders were empty, and test mice were placed in the apparatus to habituate for 10 min. Mice were then injected with saline or baclofen and returned to their home cages for 5 min. For the interaction test, a female A/J stimulus mouse was placed in one mesh cylinder and an inanimate object (tennis ball) was placed in the other. Test mice were then reintroduced into the testing apparatus and the experimenter left the room. Social interaction was quantified as the number of sniffing bouts at the mesh cylinder containing the stimulus mouse (social) versus the inanimate object (nonsocial). The number of social and nonsocial interactions was scored manually and expressed as a percentage of the total number of interactions. Seven mice (three female WT and four male WT) were assessed in the vehicle condition but not included in further analyses because of technical difficulties with recording during one baclofen test session.

Working memory was assessed using the spontaneous alternation T-maze. Mice were acclimated in white light in a room adjacent to the testing room for 15 min before recording. Five minutes after injection of saline or baclofen, mice were placed at the end of the central arm of an opaque plastic T-maze (arm dimensions 30 cm long, 10 cm wide, and 12 cm tall), which contained 1 cm of fresh bedding. Navigation of the mouse in the maze was recorded for 5 min with a Sony NightShot digital camera. Arm entries were scored manually, with an entry recorded if the mouse progressed more than halfway into the arm and a correct alternation recorded if the entry occurred in the most novel (least recently visited) arm. The number of correct entries, as a percentage of total entries during the 5 min test, was scored manually. One male WT mouse was assessed in the vehicle but did not complete the test at 1 mg/kg baclofen and was excluded. Testing took place in a dimly lit room.

Finally, episodic memory was assessed on a modified radial water maze task. The apparatus consisted of four transparent plastic arms (arm dimensions 30 cm long, 10 cm wide, and 24 cm tall), with a 20 cm square central zone contained in a room under white light with distal visual cues (wall posters). The maze was filled to a depth of 1 cm with an opaque mixture of room temperature water and white tempura paint. This depth of water was sufficient to motivate the mice to find the platform and also to hide the platform. At the end of one arm, a white platform was placed level with the water surface. Mice were trained for three consecutive days, and on each day were given five trials (training trials) to locate the platform. Mice were gently shepherded to the platform if they were unable to locate the platform after 60 s. Mice were dried and allowed to recuperate for 15 min on a heated home cage-like enclosure between trials. On each sequential trial, the arm in which the mouse started the test was varied. Test days commenced 48 h after the last training day. Each mouse was habituated to the testing room for 10 min, injected IP with saline or baclofen in its home cage, and commenced testing (test trials) 5 min later. Mice were tested in each arm in random order, and the latency to find the platform recorded manually. The latency across the three test trials was averaged. Three mice (one female WT, one male WT, one male KO) did not complete the test on one of the baclofen testing days and were included in the vehicle, but not baclofen, analysis.

### Statistical analysis

Data from each test were approximately normally distributed (skewness between –1 and 1) with the exception of open field percentage center time and radial water maze latency, for which all data were log transformed to achieve normality. Analyses were conducted in two stages. First, analyses of genotype and sex differences were performed in the vehicle condition using factorial ANOVA. Second, main effects of genotype, sex, and baclofen dose (within-subject factor) were assessed across baclofen conditions using repeated-measures ANOVA. Post hoc comparisons were performed using the least-significant difference test when significant main effects or interactions were found. Correlations between continuous variables were investigated using Pearson’s correlations. No outliers were removed. All analyses were performed using Statistica (Dell Inc.; RRID: SCR_014213).

## Results

### Effects of *Fmr1* KO and baclofen on ERP measures

#### P20

**Figure 1.**
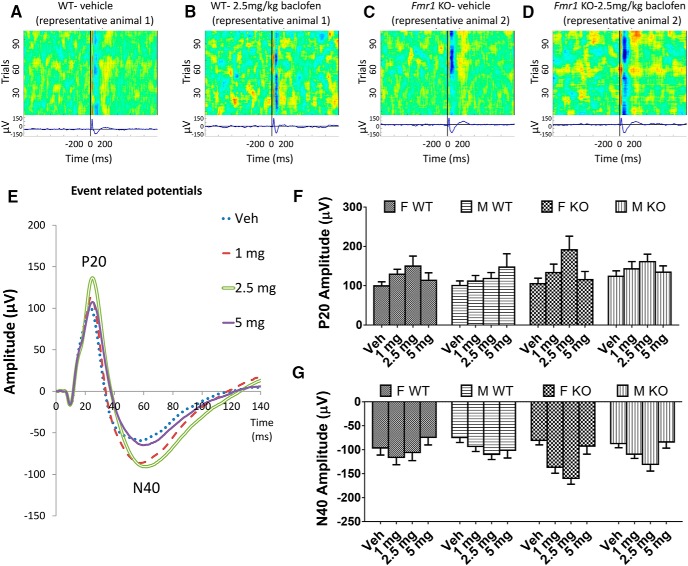
Effects of baclofen on ERPs in *Fmr1* KO and WT mice (F-WT, *n* = 16; F-KO, *n* = 15; M-WT, *n* = 23; M-KO, *n* = 18). ***A–D***, Heat maps and traces showing positive and negative ERP deflections across test trials from representative WT (***A***, ***B***) and *Fmr1* KO (***C***, ***D***) mice after vehicle and 2.5 mg/kg baclofen administration. ***E***, Grand average waveforms with characteristic ERP deflections in all mice (female and male *Fmr1* KO and WT) after vehicle or 1, 2.5, and 5 mg/kg baclofen. ***F***, Effects of baclofen on amplitudes of the P20 component of the ERP in female and male *Fmr1* KO and WT mice. ***G***, Effects of baclofen on amplitudes of the N40 ERP component. F, female, M, male, Veh, vehicle. Error bars represent SEM.

Amplitude of the P20 did not differ between *Fmr1* KO and WT mice (*p* = 0.26; Fig. 1*A*, *C*, *F*) nor between male and female mice (*p* = 0.45; Fig. 1*F*) in the vehicle condition. Baclofen increased P20 amplitude at all doses [*F*_(3,204)_ = 8.30, *p* = 0.00003; Fig. 1*B*, *D–F*], with the greatest effect at 2.5 mg/kg. Across baclofen doses, P20 amplitude did not differ across genotypes or sexes (*p* = 0.31 and *p* = 0.98 respectively; Fig. 1*F*). The effect of baclofen on P20 amplitude did not differ between *Fmr1* KO and WT mice (genotype × drug interaction, *p* = 0.11) but was larger in female than male mice [sex × drug interaction, *F*_(3,204)_ = 3.14, *p* = 0.026; Fig. 1*F*].

#### N40

A similar pattern was observed with N40 amplitude, with no significant main effects of genotype or sex with vehicle (genotype, *p* = 0.86; sex, *p* = 0.51; Fig. 1*A*, *C*, *G*) or during baclofen treatment (genotype, *p* = 0.11; sex, *p* = 0.30; Fig. 1*B*, *D*, *G*). There was a significant main effect of baclofen [*F*_(3,204)_ = 11.75, *p* < 0.000001; Fig. 1*E*, *G*]. N40 amplitude was increased at 1 mg/kg (*p* = 0.001) and 2.5 mg/kg (*p* = 0.000002) baclofen relative to vehicle, but not at 5 mg (*p* = 0.53). The effect of baclofen on N40 amplitude did not differ between *Fmr1* KO and WT mice (genotype × drug interaction, *p* = 0.07) or between female and male mice (drug × sex interaction, *p =* 0.23).

### Effects of *Fmr1* KO and baclofen on high-frequency neural oscillations

#### Baseline power

**Figure 2.**
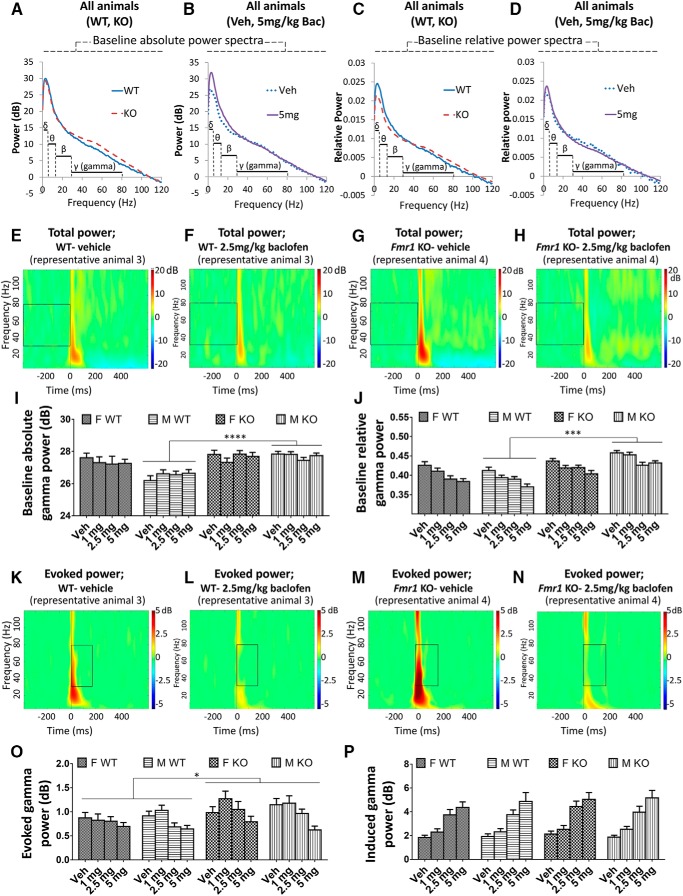
Effects of baclofen on baseline and auditory stimulus–related gamma oscillations in *Fmr1* KO and WT mice (F-WT, *n* = 16; F-KO, *n* = 15; M-WT, *n* = 23; M-KO, *n* = 18). ***A***, ***B***, Baseline absolute power spectra in *Fmr1* KO and WT mice (***A***), and vehicle and 5 mg/kg baclofen treatment conditions (***B***). ***C***, ***D***, Baseline relative power spectra (normalized to total 0 to 120 Hz power). ***E–H***, Heat maps showing total power of EEG oscillations at baseline across 2 to 120 Hz frequencies from representative WT (***E***, ***F***) and *Fmr1* KO (***G***, ***H***) mice after vehicle and 2.5 mg/kg baclofen administration. Boxes highlight baseline gamma oscillations. ***I***, Baseline absolute gamma power. ***J***, Baseline relative gamma power. ***K–N***, Heat maps showing evoked power of EEG oscillations after stimulus across 2 to 120 Hz frequencies from representative WT (***K***, ***L***) and *Fmr1* KO (***M***, ***N***) mice after vehicle and 2.5 mg/kg baclofen administration. Boxes highlight auditory-evoked gamma oscillations. ***O***, Evoked gamma power. ***P***, Induced gamma power. F, female, M, male, Veh, vehicle. Error bars represent SEM. *p < 0.05, ***p < 0.0005, ****p < 0.00005.

The absolute power of gamma (30–80 Hz) frequency neural oscillations (Fig. 2*A*, *B*, *E–I*), and the relative power of gamma oscillations [as a proportion of total (0–120 Hz) power; Fig. 2*C*, *D*, *J*] were quantified, because oscillations at this frequency are thought to relate to cognitive function (Salinas and Sejnowski, 2001; Buzsáki and Wang, 2012) as well as being driven by the activity of GABAergic interneurons (Buzsáki and Wang, 2012) that are implicated in FXS (Adusei et al. 2010; Paluszkiewicz et al. 2011). For baseline absolute gamma power in the vehicle condition, there was greater power in *Fmr1* KO than in WT mice overall [*F*_(1,68)_ = 11.73, *p* = 0.001; Fig. 2*I*]. This genotype difference was present in males (*p* = 0.00002; Fig. 2*I*) but not in females (*p* = 0.61) [genotype × sex interaction: *F*_(1,68)_ = 7.07, *p =* 0.013]. Across all baclofen doses, the overall genotype difference was still evident [*F*_(1,68)_ = 11.34, *p* = 0.001]. Again, this genotype difference trended toward a significant difference in males (*p* = 0.0001; Fig. 2*I*) but not females [*p* = 0.35; genotype × sex interaction, *F*_(1,68)_ = 3.85, *p* = 0.054]. In the vehicle condition, there was greater baseline absolute gamma power in females than males [*F*_(1,68)_ = 6.44, *p* = 0.013], driven by sex differences in WT mice (p = 0.00002; Fig. 2*I*) but not *Fmr1* KO mice (*p* = 0.93) owing to the genotype × sex interaction. There was no effect of baclofen on baseline absolute gamma power (*p* = 0.76; Fig. 2*I*).

For baseline relative gamma power after vehicle, there was also greater power in *Fmr1* KO mice than in WT mice [*F*_(1,68)_ = 10.99, *p* = 0.001; Fig. 2*J*] and a trend toward a significant genotype sex interaction [*F*_(1,68)_ = 3.89, *p* = 0.053] with an effect of genotype in males (*p* = 0.0002; Fig. 2*J*) but not females (*p* = 0.38). This genotype effect held across baclofen treatment conditions [*F*_(1,68)_ = 37.06, *p <* 0.000001], and as seen in vehicle, male *Fmr1* KO mice had greater elevations of relative baseline gamma power compared with WT than females [genotype × sex interaction, *F*_(1,68)_ = 9.23, *p* = 0.003; Fig. 2*J*]. In contrast to absolute gamma power, there was no overall sex difference (vehicle, *p* = 0.56; baclofen, *p* = 0.26). Baseline relative gamma power was strongly decreased by baclofen [*F*_(3,204)_ = 27.21, *p* < 0.000001; Fig. 2*J*].

#### Auditory stimulus–related power

Changes to gamma power in response to auditory stimuli were also investigated (Fig. 2*K–N*). In the vehicle condition, the absolute power of evoked gamma oscillations, which are time-locked to the stimulus, did not differ between genotypes (*p* = 0.15; Fig. 2*O*). However, across all baclofen treatment groups together, evoked gamma power was significantly increased in *Fmr1* KO mice compared with WT mice [*F*_(1,68)_ = 5.28, *p* = 0.025; Fig. 2*O*]. There were no sex differences in evoked gamma power (vehicle, *p* = 0.39; baclofen, *p* = 0.88). A significant decrease in evoked gamma power was observed after baclofen [*F*_(3,204)_ = 13.96, p < 0.000001; Fig. 2*K–N*, *O*], which was strongest at 5 mg/kg (*p* = 0.000003) relative to vehicle. A marginal effect was also observed at 2.5 mg/kg (*p* = 0.054). Baclofen had the same effect in WT and *Fmr1* KO mice (genotype × drug interaction, *p =* 0.17) and across sexes (sex × drug interaction, *p* = 0.21; Fig. 2*O*). The power of induced gamma oscillations, which are not time-locked to the stimulus, was not influenced by genotype (vehicle, *p* = 0.59; baclofen, *p* = 0.35; Fig. 2*P*) or sex (vehicle, *p* = 0.69; baclofen, *p* = 0.99). However, induced gamma power was strongly increased by baclofen at all doses [*F*_(3,204)_ = 73.59, *p* < 0.000001; Fig. 2*P*]. This effect was equivalent in *Fmr1* KO and WT mice (genotype × drug interaction, *p =* 0.81) and female and male mice (sex × drug interaction, *p* = 0.67).

### Effects of *Fmr1* KO and baclofen on behavior

**Figure 3.**
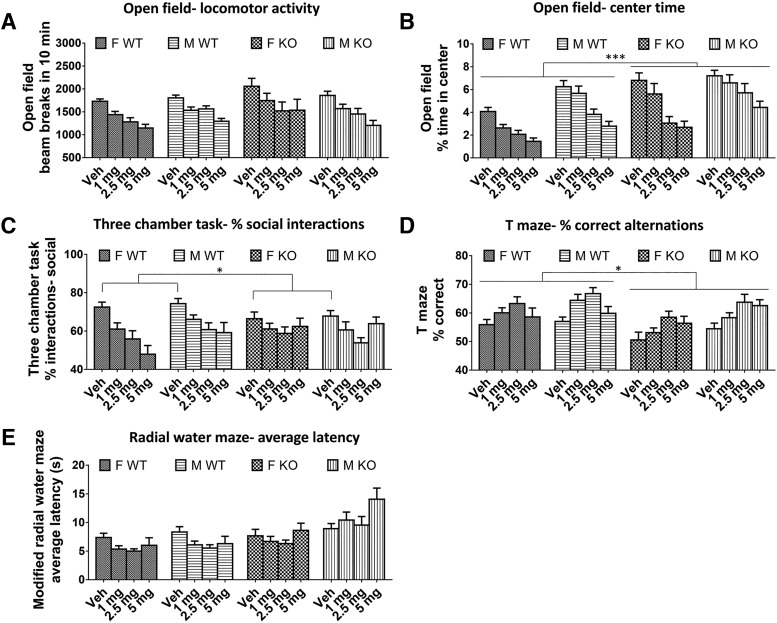
Effects of baclofen on behaviors in *Fmr1* KO and WT mice. ***A***, Open-field beam breaks in 10 min; F-WT, *n* = 18; F-KO, *n* = 18; M-WT, *n* = 22; M-KO, *n* = 19. ***B***, Percentage center time spent in the open field. ***C***, Percentage of social interactions in the three-chamber test; F-WT, *n* = 15; F-KO, *n* = 15; M-WT, *n* = 18; M-KO, *n* = 19. ***D***, Percentage of correct spontaneous alternations in the continuous T-maze task; F-WT, *n* = 17; F-KO, *n* = 18; M-WT, *n* = 22; M-KO, *n* = 19. ***E***, Average latency to find the platform during test trials in the modified radial water maze; F-WT, *n* = 18; F-KO, *n* = 18; M-WT, *n* = 18; M-KO, *n* = 18. F, female, M, male, Veh, vehicle. Error bars represent SEM. *p < 0.05, ***p < 0.0005.

There was no difference between *Fmr1* KO and WT mice in the number of beam breaks in 10 min with vehicle (*p* = 0.33) or during baclofen treatment (*p* = 0.28; Fig. 3*A*) in the open-field test. There was also no effect of sex (vehicle, *p* = 0.67; baclofen, *p* = 0.77). However, baclofen significantly decreased locomotor activity at all doses [*F*_(3,213)_ = 39.38, *p* < 0.000001; Fig. 3*A*] in both genotypes and sexes (genotype × drug interaction, *p* = 0.52; sex × drug interaction, *p* = 0.44). For center time in the open field, there were significant main effects of genotype, sex, and baclofen treatment. *Fmr1* KO mice spent a significantly greater proportion of the total time in the center than WT mice [vehicle: *F*_(1,73)_ = 17.70, *p* = 0.00007; baclofen: *F*_(1,71)_ = 12.49, *p* = 0.0007; Fig. 3*B*]. Males spent significantly more time in center than females [vehicle: *F*_(1,73)_ = 7.62, *p* < 0.007; baclofen: *F*_(1,71)_ = 28.68, *p =* 0.000001; Fig. 3*B*]. Baclofen significantly decreased percentage center time [*F*_(3,213)_ = 50.11, *p* < 0.000001; Fig. 3*B*], reversing the *Fmr1* KO phenotype. This effect was similar in both genotypes (genotype × drug interaction, *p* = 0.51) but was greater in females [sex × drug interaction, *F*_(3,213)_ = 3.23, *p <* 0.05; Fig. 3*B*].

Both *Fmr1* KO and WT mice displayed a preference for interactions with the target mouse rather than the object (percentage social interactions >50%; Fig. 3*C*) during the three-chamber social interaction test. In the vehicle condition, social preference was decreased in *Fmr1* KO mice compared with WT [*F*_(1,63)_ = 4.58, *p =* 0.036; Fig. 3*C*]. However, during baclofen treatment, this main effect was not significant (*p* = 0.87). There was no difference between males and females following either vehicle or baclofen (vehicle, *p* = 0.59; baclofen, *p* = 0.28). Baclofen significantly reduced sociability [*F*_(3,168)_ = 10.43, *p =* 0.000002; Fig. 3*C*] to a similar extent in both genotypes (genotype × drug interaction, *p* = 0.11) and sexes (sex × drug interaction, *p* = 0.70).

Significant main effects of genotype, sex, and treatment were found on spontaneous alternation in the T-maze. *Fmr1* KO mice had a significantly lower proportion of correct alternations than WT mice across baclofen doses [*F*_(1,70)_ = 5.84, *p* = 0.018; Fig. 3*D*]. This was less evident in the vehicle condition, which failed to reach significance (*p* = 0.057). Males made more correct alternations than females across baclofen doses [*F*_(1,70)_ = 9.16, p = 0.003], but this was not significant in the vehicle condition alone (*p* = 0.22). The working memory of all mice was significantly improved by baclofen [*F*_(3,210)_ = 10.71, *p =* 0.000001; Fig. 3*D*], with no significant genotype × drug (*p* = 0.10) or sex × drug (*p* = 0.84) interactions.

No effects of genotype or sex on latency to find the platform were observed on the radial water maze during test trials following vehicle (genotype, *p* = 0.67; sex, *p* = 0.17). However, across baclofen doses, significant main effects were found for genotype [*F*_(1,65)_ = 21.70, *p =* 0.00002; Fig. 3*E*] and baclofen [*F*_(3,195)_ = 3.55, *p* = 0.015; Fig. 3*E*], which were moderated by a genotype × drug interaction [*F*_(3,195)_ = 3.56, *p =* 0.015]. The main effect of genotype arose because there were contrasting effects of baclofen in WT and *Fmr1* KO mice (Fig. 3*E*). In WT mice, baclofen at all doses decreased latency compared with vehicle (all at least *p* < 0.05), whereas in *Fmr1* KO mice, baclofen had no significant effect. Finally, across baclofen doses, latency to approach the platform was lower in female mice than male mice [*F*_(1,65)_ = 8.64, *p* = 0.005].

### Relationship between baclofen gamma response and T-maze performance improvement

To explore the plausibility of using EEG to predict behavioral treatment responsiveness, we investigated whether rescue of specific EEG abnormalities (baseline relative gamma power and evoked gamma power) at an individual level was associated with improvement in T-maze spontaneous alternation or open-field center time in *Fmr1* KO and WT mice. These parameters were investigated at 2.5 mg/kg baclofen, the dose at which most consistent improvement of electrophysiological and behavioral deficits in *Fmr1* KO mice was seen.

**Figure 4.**
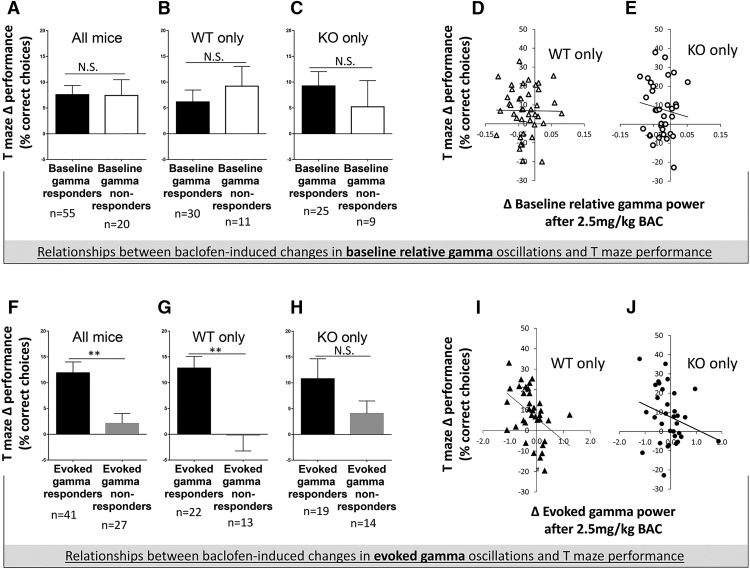
Relationships between changes in gamma power and improvements in T-maze performance after 2.5 mg/kg baclofen. ***A–C***, Differences in T-maze performance between baseline relative gamma responders and baseline relative gamma nonresponders. ***D***, ***E***, Correlation of changes in relative baseline gamma power after 2.5 mg/kg baclofen with changes in T-maze performance in WT and *Fmr1* KO mice. ***F–H***, Differences in T-maze performance between evoked gamma responders and evoked gamma nonresponders. ***I***, ***J***, Correlation of changes in evoked gamma power after 2.5 mg/kg baclofen with changes in T-maze performance in WT mice and *Fmr1* KO mice. BAC, baclofen, N.S., not significant. Error bars represent SEM. **p ≤ 0.005.

First, mice were grouped into baseline-relative gamma responders, whose baseline relative gamma power decreased with baclofen (in *Fmr1* KO mice, this represented rescue of their abnormal baseline relative gamma power), and baseline relative gamma nonresponders, whose baseline relative gamma power increased with baclofen. There was no difference in Δ T-maze performance (the change in percentage correct alternations after 2.5 mg/kg baclofen treatment compared with vehicle) between baseline relative gamma responders and nonresponders (all mice, *p* = 0.79; WT only, *p* = 0.26; *Fmr1* KO only, *p* = 0.51; Fig. 4*A–C*). There was also no correlation of Δ baseline relative gamma power with Δ T-maze performance in WT or *Fmr1* KO mice (WT only, *p* = 0.77; *Fmr1* KO only, *p* = 0.50; Fig. 4*D*, *E*]. Second, mice were grouped into evoked gamma responders, whose evoked gamma power decreased with baclofen (in *Fmr1* KO mice, this represented rescue of their deficit), and evoked gamma nonresponders, whose evoked gamma power increased with baclofen. In contrast to baseline gamma, there was a significant difference in postbaclofen T-maze performance between evoked gamma responders and nonresponders (Fig. 4*F–H*). These effects reached significance in all mice together (*t* = 3.16, df = 66, *p* = 0.003; Fig. 4*F*) and in WT mice (*t* = 3.40, df = 33, *p* = 0.002; Fig. 4*G*), but failed to do so in *Fmr1* KO mice (*t* = 1.35, df = 31, *p* = 0.19; Fig. 4*H*).There was an inverse correlation between Δ evoked gamma power responses and Δ T-maze performance in all mice (*r* = –0.33, *p* = 0.006) and in WT mice (*r* = –0.43, *p* = 0.010; Fig. 4*I*) but not in *Fmr1* KO mice (*r* = –0.26, *p =* 0.14; Fig. 4*J*). Collectively, the animals that displayed the greatest baclofen-induced decrease in evoked gamma power showed the greatest improvement in T-maze performance. None of the above relationships were evident for open-field center time, in that there were no relationships between changes in baseline relative gamma power or evoked gamma power and open-field center time after 2.5 mg/kg baclofen (data not shown). Similarly, there were no significant correlations between changes in induced power and behavior after 2.5 mg/kg baclofen, or between changes in EEG measures and sociability (data not shown).

## Discussion

After mixed results in human and animal studies, it is unclear whether baclofen and other pharmacotherapies targeting GABAergic neurotransmission can effectively treat the symptoms of FXS, particularly in the absence of biomarker-based strategies to target treatment to subsets of individuals. In this study, we present evidence that (a) sensory hypersensitivity occurs in *Fmr1* KO mice, as indexed by increased evoked EEG gamma power, (b) baclofen normalizes this increased evoked EEG gamma power and improves working memory (T-maze performance), and (c) at an individual level, normalization of evoked gamma oscillations was associated with improvement in working memory. Our study also suggests that some *Fmr1* KO–related deficits, such as impaired sociability and episodic memory (radial water maze performance), may be impervious to rescue by baclofen. Overall, these findings indicate that racemic baclofen may have benefit for remediating sensory hypersensitivity and some cognitive deficits in FXS and suggest that evoked gamma oscillations may have potential utility for prediction of treatment responsiveness in some behavioral domains in the condition.

Increased evoked gamma power in *Fmr1* KO mice is consistent with sensory hypersensitivity (Karakaş and Başar, 1998), which is also seen in *Fmr1* KO mice in the form of diminished ERP N1 habituation (Lovelace et al. 2016), neuronal hyperexcitability in the auditory cortex (Rotschafer and Razak, 2014), and increased prepulse inhibition (Chen and Toth, 2001). Increased sensitivity to sensory stimuli is also often observed in individuals with FXS (Kogan et al. 2004; Baranek et al. 2008). The diminution of evoked gamma power by racemic baclofen suggests that this agent may be particularly valuable in targeting the specific sensory hypersensitivity endophenotype in FXS.

A key finding of this study was that changes in evoked gamma power after 2.5 mg/kg baclofen were related to changes in T-maze performance, such that evoked gamma responders showed greater T-maze performance improvement than nonresponders. Baclofen-induced EEG changes, rather than predrug EEG measures, were analyzed because of their greater clinical potential. They enable a within-subject absolute criterion (evoked gamma decrease, yes/no) to be used to classify individual subjects and do not require normative reference data. In contrast, use of predrug evoked gamma power (analogous to vehicle treatment) would involve determination of normative ranges and collection of extensive reference data. Between genotype groups, there was a less strong relationship between baclofen-induced changes in evoked gamma oscillations and T maze performance in *Fmr1* KO mice than in WT mice. This was expected, since baclofen targets a neurotransmitter system known to be dysregulated in *Fmr1* KO mice. However, our findings suggest that further preclinical study of the possible link between evoked power changes and drug effectiveness for other pharmacotherapies is warranted given the potential benefit of this approach for individuals with FXS.

Increased baseline gamma power in FXS is a robust feature of the disorder, indicative of increased network noise and network hyperexcitability (Gandal et al. 2012a). It is observed in humans with FXS (Ethridge et al. 2016) and ASD (Orekhova et al. 2007; Wang et al. 2013). It is also seen in *Fmr1* KO mice *in vivo* (in this study) and *in vitro* in thalamocortical slices from *Fmr1* KO mice (Gibson et al. 2008). Racemic baclofen did not alter absolute power of baseline gamma oscillations in our study, in contrast to a prior study in which l-baclofen decreased absolute power of oscillations between 21 and 50 Hz (Marrosu et al. 2006). However, baclofen did decrease the relative power of baseline gamma oscillations as a proportion of total oscillatory activity. Further work is required to determine whether the importance of high-frequency oscillations lies in their absolute power or their power relative to other oscillatory activity. If absolute gamma power is more neurobiologically relevant, the failure of baclofen to rescue increased absolute baseline gamma power in *Fmr1* KO mice may represent a limitation of this pharmacotherapy. If relative power of baseline oscillations has particular relevance, then investigation of other frequencies in *Fmr1* KO mice is warranted, given that differences in relative upper alpha (10–12 Hz) and theta (4–8 Hz) power have been described in FXS (Van der Molen and Van der Molen, 2013). Nonetheless, as a potential translational biomarker, baseline gamma power may be a useful tool for designing and evaluating future therapeutic strategies for FXS.

The behavioral effects of baclofen reported here are partially consistent with prior studies. Improvement of spatial working memory by systemic baclofen has previously been reported in a similar T-maze task (Gandal et al. 2012b) and is in line with other cognition-enhancing properties of baclofen, including increased behavioral flexibility in rats (Beas et al. 2016). Because loss of FMRP results in abnormally increased open-field center exploration, it has been argued that the decrease in open-field center time by baclofen represents a beneficial, anxiety-normalizing effect (Bhogal and Jongens, 2010). Interestingly, this effect was not seen with arbaclofen at 1.5 mg/kg (Qin et al. 2015). Together with these beneficial effects of baclofen, there were indications of other effects that may not be therapeutically helpful. In particular, baclofen decreased sociability at all doses. This contrasts with two previous studies, one that found a beneficial effect of baclofen on sociability (Gandal et al. 2012b) and another that found no effect, but reported an increase in preference for social novelty (preference for novel over familiar stimulus mouse; Qin et al. 2015).

Although *Fmr1* KO mice effectively mimic the loss of FMRP protein in FXS, they do not display a highly consistent behavioral phenotype. Some studies have reported spatial memory deficits in *Fmr1* KO mice (Bakker et al. 1994; D’Hooge et al. 1997), but others have not (Peier et al. 2000; Leach et al. 2016). Deficits in working and episodic memory in *Fmr1* KO mice are consistent with the clinical picture in FXS, in which intellectual disability is commonly seen. Decreased anxiety-like behavior and hyperactivity in the open field in *Fmr1* KO mice are very often observed (e.g., Uutela et al. 2012; Dolan et al. 2013; Ding et al. 2014) but not always (Veeraragavan et al. 2011). A possible reason for this discrepancy is that the hyperactivity phenotype may diminish with repeated testing (Kramvis et al. 2013). Decreased social interactions by *Fmr1* KO mice have been reported in free-interacting tests (Hébert et al. 2014; Oddi et al. 2015). However, when the three-chamber social interaction test is used, abnormalities are often not seen in sociability (Liu et al. 2011; Hébert et al. 2014), although other work has even shown increased sociability in *Fmr1* KO mice (Gantois et al. 2013).

A possible reason for this inconsistency across studies with *Fmr1* KO mice could involve stress, since *Fmr1* KO mice appear to show enhanced corticosterone secretion in response to acute stress (Markham et al. 2006; Ghilan et al. 2015, but see Qin and Smith, 2008). The differential effects of acute stress on *Fmr1* KO compared with controls may contribute to the *Fmr1* KO behavioral phenotype in some tests, such as the T-maze or radial arm maze. Consequently, this phenotype may be particularly impacted by differences in the testing environment between studies. Furthermore, the animals in this study were individually housed after surgery and are likely to have experienced some longer-term stress associated with sustained social isolation that was not experienced by animals in other studies. Heightened adaptive or maladaptive responses of *Fmr1* KO mice to chronic stress (Qin et al. 2011) may have further influenced genotype effects in this study. Future studies are warranted to clarify the role of stress in *Fmr1*-related pathology in mice and determine whether social isolation or socialization impacts the expression of functional deficits in humans with FXS.

Sex × genotype interactions were observed for absolute and relative baseline gamma power, with increased baseline gamma power seen in male but not female *Fmr1* KO mice. This is consistent with the increased severity of FXS in human males (Crawford et al. 2001). However, unlike in humans, sex differences in the expression of FXS-related phenotypes in this study are unlikely to relate to variable penetrance of the genetic manipulation/mutation, since this study used complete knockout of *Fmr1* for both sexes. Instead, they are more likely to reflect the interaction of FMRP signaling with sex differences in other pathways.

Several lines of evidence have identified GABAergic interneurons as the primary source of gamma oscillations in the brain (Traub et al. 1996; Bartos et al. 2007). Mice with the NMDA subunit 1 receptor selectively removed from parvalbumin-expressing GABAergic interneurons show a similar EEG profile to *Fmr1* KO mice, including increased basal gamma EEG, and display several autism-like behavioral changes (Carlén et al. 2012; Saunders et al. 2013; Billingslea et al. 2014). Increased gamma oscillations are thought to be due to a loss of the normal inhibitory control typically exerted by GABAergic cells, leading toward a loss of excitatory/inhibitory balance. This suggests that the primary pathology underlying the EEG abnormalities reported here in *Fmr1* KO mice may reflect a loss of GABA integrity. Baclofen stimulates GABA-B receptors both presynaptically, resulting in decreased glutamate release (Isaacson and Hille, 1997), and postsynaptically, resulting in decreased excitability due to voltage gated potassium channel activation (Lüscher et al. 1997). GABA-B agonism by baclofen may compensate for GABAergic pathology in *Fmr1* KO mice, such as decreased GABA receptor abundance (Gantois et al. 2006; Adusei et al. 2010). Additionally, arbaclofen has also been shown to decrease protein synthesis (Henderson et al. 2012; Qin et al. 2015), potentially counteracting excess protein production due to FMRP loss (Darnell et al. 2011).

This study had a number of limitations. A within-subject design was used, enabling assessment of the acute effects of baclofen across multiple domains. However, later behavioral tests (such as social interaction and radial water maze) may have been influenced by lingering effects of prior baclofen exposure despite the 4 to 5 d washout. In the open-field test and other tests, habituation to the test environment may also have modified the behavioral measures and confounded the effects of baclofen. Furthermore, findings at the highest baclofen dose (5 mg/kg) may have been influenced by the sedative effect of the drug, accounting for its diminished efficacy in T-maze and water maze tests at that dose. Ultimately, the value of the findings described in this study will be determined by the extent of their translatability and predictive validity for individuals with FXS.

## Synthesis

Reviewing Editor: Cheryl Sisk, Michigan State University Decisions are customarily a result of the Reviewing Editor and the peer reviewers coming together and discussing their recommendations until a consensus is reached. When revisions are invited, a fact-based synthesis statement explaining their decision and outlining what is needed to prepare a revision will be listed below. The following reviewer(s) agreed to reveal their identity: Andre Fenton, Jay Gibson

Because your manuscript was transferred from Journal of Neuroscience and you had revised the manuscript in response to the JN reviews, I asked the two original reviewers to take a look at the revised manuscript and determine whether their concerns had been addressed. For the most part, they were, but there are a few additional comments, and I ask you to respond them and make further revisions as appropriate.

1) One reviewer noted a number of grammatical errors that should be fixed, but did not provide specifics. Please check your manuscript carefully and correct any errors that you find.

2) The figure referencing should be checked for accuracy. For example: page 13 cites Fig. 2A,B, E-G for base line gamma - shouldn't this include H?

Page 14 top: Fig. 2F is cited for a sex difference, which doesn't make sense to me.

3) The authors are overly liberal in what they accept as significant ANOVA effects, which they assert is alpha <= 0.05. However, they accept p = 0.053 and perform subsequent post-hoc tests in the middle paragraph of page 14. At the top of page 15 they accept p = 0.054 as significant. I am not asking that they do anything radical except adhere to their own criteria and report the effects as marginal but not significant.

4) The correlation between change in evoked gamma responses to baclofen and the changed T-maze alternation is very interesting. Although the rationale for the correlations that are presented is sound, it would be good to know that that they examined the correlations with other tasks that the drug affected even if it was not normalizing like sociability, and also use induced gamma as a predictor. Just reporting in the Discussion whether or not any of these relationships were significant will be welcome and informative.

5) The Discussion is generally good and comprehensive and while it discusses gamma and how baclofen might act, they never explain the well-accepted biophysical GABAergic origins of gamma. This omission undermines the findings as it appears that gamma is an abstract phenomenon with no physiological basis.

6) Although they only mention that episodic memory was assessed in the Discussion, it is not obvious what task(s) assessed episodic memory. Was it T-maze alternation, or the radial arm maze? Both have been interpreted to measure working memory and episodic memory. It is simply not clear what findings the authors are referencing when they revert to such terms.

7) Page 21: The authors discuss that baclofen's action could be indirect such as by affecting sleep. While this is true in general it seems not to apply to the present experiments because the acute immediate effects of baclofen were assessed. That needs to be pointed out otherwise the Discussion point is misleading.

8) I do not understand the logic of the argument that baclofen is acting on a FMRP-independent mechanism in the Discussion that starts on page 20 and continues on 21. This seems obvious given the mechanism of action of baclofen, and the argument seems both unnecessary and sophist as it seems to me the same argument applies for anything that that does not mimic FMRP's action, such as bind the same RNA's that FMRP binds. After all baclofen was not invented or designed for FXS.
